# Book received

**DOI:** 10.1155/S1463924601000074

**Published:** 2001


					Book received
Pharmaceutical Substances, syntheses, patents, applications. Edi-
ted by Axel Kleeman and Ju
 rgen Engel (Georg Thieme
Verlag, Stuttgart, November 2000), 2 volumes, 2672 pp.,
DM998, O
 S7285, SFr888 (hbk), ISBN 313 5584046/655.
. Alphabetical organization of all compounds accord-
ing to INN standards. Full patent information,
including application and expiry dates.
Commercial information: name of company that
markets the compound and trade name.
. Additional indexes for intermediates, trade names,
enzymes, micro-organisms, plant and animal tis-
sues, and substance classes.
Journal of Automated Methods & Management in Chemistry, Vol. 23, No. 2 (March-April 2001) pp. 67
Journal of Automated Methods & Management in Chemistry ISSN 1463 9246 print/ISSN 1464 5068 online # 2001 Taylor & Francis Ltd
http://www.tandf.co.uk/journals
DOI: 10.1080/14639240110037144
67

				

